# Influence of Processing Methods on Proximate Composition and Dieting of Two* Amaranthus* Species from West Cameroon

**DOI:** 10.1155/2016/6707313

**Published:** 2016-12-18

**Authors:** Arnaud Landry Suffo Kamela, Raymond Simplice Mouokeu, Rawson Ashish, Ghislain Maffo Tazoho, Lamye Glory Moh, Etienne Pamo Tedonkeng, Jules-Roger Kuiate

**Affiliations:** ^1^Department of Biochemistry, Faculty of Science, University of Dschang, P.O. Box 67, Dschang, Cameroon; ^2^Department of Food Engineering and Design, Indian Institute of Crop and Processing Technology, Thanjavur, India; ^3^Institute of Fisheries and Aquatic Sciences, University of Douala, P.O. Box 7236, Douala, Cameroon; ^4^Department of Animal Production, Faculty of Agronomy and Agricultural Science, University of Dschang, Dschang, Cameroon

## Abstract

The effects of various processing methods on the proximate composition and dieting of* Amaranthus hybridus* and* Amaranthus cruentus* from West Cameroon were investigated in this study. Both amaranths leaves were subjected to same treatments (sun-dried and unsliced, sliced and cooked), milled, and analysed for their mineral and proximate composition. Thirty-Six* Wistar* albino rats of 21 to 24 days old were distributed in six groups and fed for 14 days with 10% protein based diets named D0 (protein-free diet), DI (egg white as reference protein), DII (sun-dried and unsliced* A. hybridus*), DIII (cooked and sliced* A. hybridus*), DIV (sun-dried and unsliced* A. cruentus*), and DV (cooked and sliced* A. cruentus*). The protein bioavailability and haematological and biochemical parameters were assessed in rats. The results showed that K, P, Mg, Zn, and Fe had the higher content in both samples regardless of processing method. The sun-dried and unsliced* A. cruentus* contained the highest value of crude protein 32.22 g/100 g DM (dry matter) while the highest crude lipid, 3.80 and 2.58%, was observed, respectively, in sun-dried and unsliced* A. hybridus* and cooked and sliced* A. cruentus*. Cooked and sliced* A. hybridus* and* A. cruentus* contained high crude fiber of 14 and 12.18%, respectively. Rats fed with diet DIII revealed the best protein bioavailability and haematological parameters whereas 100% mortality rate was recorded with group fed with diet DIV. From this study, it is evident that cooked and sliced* A. hybridus* and* A. cruentus* could play a role in weight reduction regimes.

## 1. Introduction

Vegetables and fruits offer the most rapid and cheapest sources of adequately supplied vitamins, minerals, and some essential amino acids [1]. They have the cheapest and most abundant sources of protein [2]. In Cameroon, as in many Africa countries, vegetables are very abundant immediately after the first rains, but they become scarce in the middle of the rainy season and more so in the dry season [3].

A great variety of local and introduced vegetable crops are grown in Cameroon and these crops together with a significant number of wild and semiwild plants like* Amaranthus hybridus *and* Amaranthus cruentus* form a valuable complementary food in the daily diet [4]. Most of them are consumed in the rural areas or in the communities where they are being planted [5]. They are underutilized when compared to the introduced varieties due to the flavor and unfamiliar taste impacted on the food [6, 7]. They are very perishable commodities with very high moisture contents; therefore, dehydration results in substantial reduction in weight and bulk with consequent savings in storage costs [8]. As green leafy vegetables are edible parts of the plants and are usually cooked before consumption, cooking causes significant changes in the nutritional properties of food as well as gelatinization of starches and coagulation of proteins to improve their digestibility and sensory properties [9]. Udosen and Ukpanah [10] observed that processing causes losses in some of the antinutritional factors and some good nutrients as well.

In many homes in Cameroon, the outstanding preservative method practiced on green leafy vegetables is slicing or not, followed by sun drying which is often combined with cooking. These methods are found to be effective in improving digestibility and increasing nutrient bioavailability and also minimize foodborne diseases [11]. Through boiling, some antinutrients contents of the leaves can be reduced [12]. However, information appears scanty on the nutritional composition of the vegetable harvested in Cameroon. The aim of this work was to determine the mineral and proximate composition of two leafy vegetables consumed in West Cameroon which undergo slicing, sun drying, and cooking processing techniques as well as protein bioavailability using rats' model. The awareness will encourage the consumption and exploitation of these cheap sources of not well-known food item at any time and throughout the year.

## 2. Materials and Methods

### 2.1. Plant Leaves Collection and Processing

The leaves of* Amaranthus hybridus* and* Amaranthus cruentus* were obtained from cultivated farmlands located at Foto, Dschang City of the West Region of Cameroon, from April 2013 to September 2013. The botanical identification was done at the National Herbarium in Yaounde (Cameroon) by referring to the voucher specimen number 15630 HNC.

The collected samples were thoroughly mixed, their stalks and dust discarded, and they were divided into two groups. The first group was sun dried without slicing with frequent turning till leaves were crumbly, while the second group (100 g) was cooked after slicing for 15 min following bleaching in boiling water (1000 mL) for 10 minutes. Cooked and sliced samples were cooled at ambient temperature (20°C ± 2), water was removed, and the sample sun dried for 3 days.

### 2.2. Assessment of Minerals and Heavy Metals Content

Minerals and heavy metals including Na, K, Ca, P, Mg, Mn, Fe, and Zn and As, Hg, Pb, and Cd, respectively, were analysed using ICP-OES (Perkin-Elmer; Model Optima™ 2000 DV, Schwerzenbach, Switzerland) [13]. Analyses were performed in triplicate.

### 2.3. Proximate Composition Analysis

The methods adopted for the proximate composition analysis were those recommended by the Association of Official Analytical Chemists [14]. Analyzed parameters included dry mater, ash content, crude fat, crude fibre, and proteins.

### 2.4. Evaluation of Protein Bioavailability

#### 2.4.1. Experimental Animals

Thirty-six* Wistar* albinos rats of both sexes, 21 to 24 days old, weighing 34–43 g were used in the experiment. These animals were bred in the Animal House of the Department of Biochemistry, University of Dschang, Cameroon, and were fed with a standard rat diet. Food and water were given to all animals used for the experiments ad libitum. Animals were maintained at room temperature (22 ± 2°C) and were handled according to standard protocols for the use of laboratory animals. The studies were conducted according to the ethical guidelines of Committee for Control and Supervision of Experiments on Animals (Registration number 173/CPCSEA, dated 28 January, 2000), Government of India, on the use of animals for scientific research.

#### 2.4.2. Experimental Design

The bioassay experiments were carried out according to AOAC [14] protocol. The animals were weighed, divided into six groups (D0, DI, DII, DIII, DIV, and DV) of six animals each, and housed individually in stainless steel screening bottom cages (permitting free dropping of faeces). Highly absorbent paper was placed under the cages to catch spilled food and to minimize contamination of faeces with urine.

The experimental diets (Table 1) containing 10% protein were prepared following ICN protocol [15]. The first group (D0) was given the N-free basal diet and the second group (DI) was fed with reference diet (egg white), while the test groups (DII, DIII, DIV, and DV) were randomly allocated to the diets containing test ingredients (sun-dried and unsliced* A. hybridus*, cooked and sliced* A. hybridus,* sun-dried and unsliced* A. cruentus,* and cooked and sliced* A. cruentus*).

#### 2.4.3. Nutritional Evaluations

The absorbed nitrogen, true digestibility (TD), feed efficiency (FE), efficiency of food utilization (EFU), protein efficiency ratio (PER), and net protein ratio (NPR) were all computed as described by Pirman et al. [16].

#### 2.4.4. Biochemical Assay

At the end of the experimental period (14 days), blood samples were collected following overnight fasting by cardiac puncture from chloroform anaesthetized rats into heparinised and nonheparinised tubes. The nonheparinised tubes were allowed to clot and were centrifuged at 3000 rpm for 15 min to obtain the serum. These sera were assayed for alanine aminotransferase (ALAT), aspartate aminotransferase (ASAT), creatinine, albumin, and urea using SPINREACT kit. The heparinised blood was used for determination of some haematological parameters including red blood cells, white blood cells, platelets, and their different indices [17, 18].

### 2.5. Statistical Analysis

Statistical analyses were performed using graph pad prism version 5.00 software. Data were analysed by one-way analysis of variance (ANOVA), followed by Bonferroni post hoc test. Results are expressed as mean ± standard deviation of replicated samples. Differences were considered significant at *P* < 0.05.

## 3. Results and Discussion

### 3.1. Results

#### 3.1.1. Minerals and Heavy Metal Content

The effects of processing treatments on mineral and heavy metals contents of two amaranths species are given in Table 2. Regardless of the processing methods, the level of K, P, and Mg remained high in both samples of sun-dried and unsliced amaranthus leaves. In both amaranths, increase of Ca, Mn, and Fe content was observed after cooking and slicing. Regardless of the amaranth, the levels of Zn and Na were relatively affected by processing methods. The levels of Hg and Cd were relatively high in* A. hybridus *regardless of the processing method.

#### 3.1.2. Proximate Composition of Leafy Vegetables

The proximate composition of protein reference (egg white) and differentially processed* A. hybridus* and* A. cruentus* are presented in Table 3. The protein content of cooked and sliced* A. cruentus* had the least value (25.65%) while the highest value (32.22%) was recorded in sun-dried and unsliced* A. cruentus* leaves sample. The various food processing techniques caused significant differences (*P* < 0.05) between sun-dried and unsliced and cooked and sliced* A. hybridus*. Cooking improved the proximate composition of the vegetables relative to the sun drying and unslicing method. However, cooking and slicing significantly decreased protein level in* A. cruentus* as well as the total lipid of* A*.* hybridus*. The ash value for* A*.* hybridus* was influenced by the processing method, with a significant increase (*P* < 0.05) in sun-dried and unsliced sample (15.43%), relative to the cooked and sliced sample (10.45 ± 0.12); meanwhile, there was no processing-related significant variation (*P* > 0.05) in the ash content of* A. cruentus*.

#### 3.1.3. Growth Performance and Protein Digestibility of Rats Fed with Processed Amaranths Leaves

The results of growth performance and protein quality on rats fed with different processed diets of* A. hybridus* and* A. cruentus* leaves are presented in Table 4. The rats fed with standard protein diet (DI) and cooked* A. hybridus* diet (DIII), respectively, had the highest food (68.56 and 73.46 g) and protein (6.74 and 7.34 g) intake and, consequently, showed the highest level of weight gain (24.4 g and 13.60 g) while those fed with cooked* A. cruentus* diet (DV) and protein-free diet (D0) significantly (*P* < 0.05) lost weight. Animals fed with sun-dried* A. hybridus* diet (DII) revealed the best feed efficiency while the highest value (16.90 g) of faeces weight was observed in animals fed with DIII. Weight of animals fed with diet DIV kept decreasing during experimental period and at the end, all the animals died. Animals fed with diet DIII significantly (*P* < 0.05) had the highest efficiency food utilization (EFU), protein efficiency ratio (PER), and net protein ratio (NPR) compared to animals fed with DI. With the death of all animals fed with DIV diet, no data about the protein quality was recorded while neither feed efficiency, EFU, and PER nor NPR were available for these animals.

#### 3.1.4. Biochemical Parameters and Haematological Indices

The results of the change in some serum enzyme activities and metabolites of rats fed with diets containing green leafy vegetables are shown in Table 5. Apart from DIII and DV, there was a significant decrease in serum activity of aspartate aminotransferase (AST) and alanine aminotransferase (ALT) of animals fed with formulated diets. The formulated diets did not influence the level of blood creatinine as compared to the control. However, these diets significantly increased (*P* < 0.05) the serum level of urea with diet DV having the highest value. On the contrary, these diets significantly decreased serum concentration of albumin with the effect of the diets being comparable. The results of the haematological parameters indicate that white blood cells (WBC), red blood cells (RBC), haemoglobin (HB), mean cell haemoglobin (MCH), mean cell haemoglobin concentration (MCHC), mean platelet volume (MPV), and plateletcrit (PCT) were not significantly different (*P* > 0.05) between diet DI and diets DII, DIII, and DV. Animals fed with diets DII, DIII, and DV showed significant decrease (*P* < 0.05) in levels of lymphocytes (LYM) and platelets (PLT) when compared to rats fed with DI. In general, there was a significant (*P* < 0.05) increase of mid-cell (MID) and granulocytes (GRAN) in groups fed with the formulated diets compared to the standard diet (DI).

## 4. Discussion

### 4.1. Mineral and Heavy Metals

The mineral analysis of treated leaves powder revealed the presence of eight (8) minerals in both samples, revealing them as excellent sources of macro- and micronutrients. The level of mineral content (K, P, and Mg) was lower in cooked and sliced leaves when compared to sun-dried and unsliced samples. This is in line with the observations of Bakr and Gawish [19], Shahnaz et al. [20], and Oboh [21] that various conventional food processing techniques (cutting, bleaching, cooking, etc.) cause a decrease in the mineral content of vegetables. Losses of the mineral elements during boiling or cooking are generally attributed to the leaching of the cell content including minerals [19]. The result of mineral analysis of the vegetables suggests consumption of enough quantities to meet Recommended Daily Allowance (RDA). The value of Na in both sun-dried and unsliced and cooked and sliced samples was low compared to the value (300.06–600.83 mg/kg) obtained by Makobo et al. [22] for sun-dried and bleached* Amaranthus cruentus*. Na is required for maintenance of fluid balance and osmotic pressure in the body for cellular activities [23, 24]. The K content found in both vegetables regardless of the processing method was higher than 241.88 mg/kg reported by Ogbadoyi et al. [25] for* Amaranthus cruentus. *However, the result of this study reveals that K content dropped below those reported in the available literature and this suggests the consumption of large quantities of these vegetables in order to meet the RDA for minerals. For instance, adult minimum K requirement for health set by the 1989 RDA is 2000 mg daily [9]. Then to meet the RDA for this important mineral involved in cellular metabolism, water used in the boiling must be included in the meal preparations [25]. The level of Ca, Mn, and Fe observed in the cooked two species of vegetables clearly indicates that these vegetables are good sources of those minerals when compared to the values obtained for cereals [26].

Calcium and phosphorus are associated with each other for the growth and maintenance of bones, teeth, and muscles [27] while Mg is an important cofactor of enzymes involved in cell respiration, glycolysis, and transmembrane transporter [23]. Iron is an essential trace element for haemoglobin formation, normal function of central nervous system, and energy metabolism [28]. The highest value of zinc (48 mg/kg) was found in sun-dried and unsliced* A. cruentus*. Therefore, the consumption of cooked and sliced* A. hybridus* in developing countries could correct Zn deficiency which is related to decreased growth in infants and children [29].

The values of As, Cd, Pb, and Hg obtained in* A. hybridus* were lower than those obtained in the same species of amaranths by Oti Wilberforce and Nwabue [30] in the state of Nigeria. The vegetables analysed regardless of the treatment methods showed lower levels of the minerals and it can be suggested that the consumption of average amounts of these vegetables could not pose a health risk for the consumers as the values obtained are far below the permissible limits of 0.2 mg/kg (cadmium), 0.1 mg/kg (arsenic), and 0.01 mg/kg (mercury) [31].

### 4.2. Proximate Composition

The results of the proximate analysis showed that sun-dried and unsliced* A. cruentus* was the richest source of crude protein, while cooked and sliced* A. hybridus* had the highest value of crude fibre. Studies have shown that these leaves are usually cooked before consumption [32]. The cooked values were therefore of great importance. The cooked and sliced sample had the lower value than sun-dried and unsliced sample. The reduction could have been due to the different levels of heat treatment and the severity of thermal process during cooking where some nutrients were leached off by water during the process [33]. Krauss et al. [34] indicated that plant foods providing more than 12% protein calorific values are good sources of protein. Therefore, the leafy vegetables studied are good sources of protein and can be used as diet supplements for people suffering from undernutrition diseases if and only if those proteins could be bioavailable. The crude fibre content in both samples ranged from 6.02 to 14% with the highest level in cooked and sliced* A. hybridus.* These values are higher than those obtained by Mensah et al. [35] in* A. cruentus* (1.8%) and Asaolu et al. [36] in* A. hybridus* (8.05%). The plants can serve as good roughage in the intestine for better functioning of the alimentary system [37] since it had been reported that food fibre aids absorption of trace elements in the gut and reduces absorption of cholesterol. Besides, vegetables rich in fibre are natural broom for the body which help to prevent constipation, bowel problems, and piles [38].

### 4.3. Growth Performance and Protein Digestibility of Rats Fed with Processed Amaranths Leaves

Weight loss was observed with the animals fed with basal diets (D0) and cooked and sliced* A. cruentus* (DV) while 100 percent of mortality rates were recorded in the group fed with sun-dried and unsliced* A. cruentus*-based diet (DIV). For the basal diet, it is not surprising since it lacks protein as Cameron and Eshelman [39] showed that deficiencies in dietary protein slow growth and delay maturation. The loss in weight observed with rats fed with sun-dried and unsliced* A. hybridus* (DII), cooked and sliced* A. hybridus* (DIII), and cooked and sliced* A. cruentus* (DV) is then a consequence of lack of full utilization and poor protein quality indices such as low values of PER, NPR, and TD. Furthermore, it could be either due to the fact that plant protein can be encased in cellulose walls, which are hard to penetrate making proteins less accessible to digestive enzymes [40], or attributed to the rich sources of dietary fibre contained in those vegetables which are known to decrease protein utilization [41]. This finding is consistent with earlier report. Agbede et al. [42] reported that vegetables proteins have lower quality protein than the animal protein, and such a diet needs to be supplemented with another protein source relatively rich in the essential amino acids. The reduction in weight gain also implies that cooked and sliced* A. cruentus* can be used in weight reduction regimens. Studies have shown that weight reduction is one of the ways of reducing coronary risk incidence, as well as managing diabetes mellitus, dyslipidemia, hypertension, and obesity [34], and is one of the strategies for improving low high density lipoprotein cholesterol (HDL-C) levels. Those results are in line with the faeces. Indeed, the faeces bulk of animals fed with these vegetables based diets was higher than those of animals fed with the standard and nitrogen-free diets. This could be explained by the abundance of fibre content in the vegetables. Cummings [43] and EFSA [44] stated that dietary fibres contribute to an increase in faecal bulk with some beneficial physiological effect like the laxative effect, capacity to decrease gastrointestinal transit time, loose stools, bloating and distension, borborygmi, abdominal discomfort, and flatus [45]. Furthermore, faeces weight is inversely related to colon cancer [43].

The death of animal fed with sun-dried and unsliced* A. cruentus*-based diet could be probably due to the presence of high amount of antinutritional factors such as trypsin inhibitors, phytate, and polyphenols that could have interfered with metabolic processes by reducing the bioavailability of nutrients [46] and limiting the digestibility of plant protein [47] and can also provoke deleterious effects on many organs [48].

### 4.4. Biochemical Parameters and Haematological Indices

There were significant increases in serum alanine aminotransferase (ALT), albumin, and aspartate aminotransferase (AST) in rats fed with diet containing cooked and sliced* A. cruentus *and sun-dried and unsliced* A. hybridus.* This indicates possible damage of some organ such as liver and heart by those vegetables. ALT is regarded to be more specific indicator of liver inflammation, while AST may be elevated in diseases of other organs such as heart and muscle diseases [49]. There was also a significant increase in the serum albumin in rats fed with diet containing cooked and sliced* A. cruentus*; this clearly indicates that* A. cruentus* may not cause liver damage since albumin is produced mainly in the liver. Except for the group fed with cooked and sliced* A. cruentus* (0.58 ± 0.038 mg·dL^−1^), the level of creatinine in other groups was higher than the normal values (0.2–0.8 mg·dL^−1^) [50]. This is an indication that the samples contained considerable amount of phytochemical compounds that may cause kidney related malfunctions.

The best RBC and HB values were observed with animals fed with diet containing cooked and sliced* Amaranthus *species. This shows that these processed leaves help in blood formation due to availability of crude protein and iron, meaning that there is no risk relative to the anaemia and related diseases with the consumption of the studied amaranth species. White blood cell count and MID cell are related to immune system and bone marrow and are indicators of the ability of an organism to eliminate infection [51]. The white blood cell of animal fed with sun-dried and unsliced and cooked and sliced* A. hybridus*-based diet was not significantly different to the reference diet, showing that the feed does not affect the immune systems of animal.

## 5. Conclusion

The present study clearly indicates that cooking method remains the best way for good utilization of these green leafy vegetables. It was also found that cooked and sliced* A. hybridus* compared to* A. cruentus* can better support growth performance. However, cooked and sliced* A. cruentus* and* A. hybridus* could be used in weight reduction regimes.

## Figures and Tables

**Table 1:** Ingredient and composition of diets.

Ingredients (g/100 g)	Diets					
	D0	DI	DII	DIII	IV	DV
Corn starch	15	15	15	15	15	15
Corn oil	5	5	5	5	5	5
Mineral complex	4	4	4	4	4	4
Vitamin complex	1	1	1	1	1	1
Cellulose	7.9	7.9	7.9	7.9	7.9	7.9
Protein source	—	13.37	32.3	31.4	30.1	38.9
Sucrose	67.1	53.73	34.8	35.7	37	28.2
Total	100	100	100	100	100	100

D0: protein-free diet, DI: egg white (protein reference) diet, DII: sun-dried and unsliced *A. hybridus* diet, DIII: cooked and sliced *A. hybridus,* DIV: sun-dried and unsliced *A. cruentus*, and DV: cooked and sliced *A. cruentus*.

**Table 2:** Minerals and heavy metals composition (mg/Kg) of two differently processed *amaranths *species.

Processing	Na	K	Ca	P	Mg	Mn	Fe	Zn	As	Hg	Pb	Cd
*A. hybridus*												
SDU	6.185	697.3	307.7	111.5	111.8	0.117	0.624	3.027	0.024	0.109	0.007	0.002
CS	3.549	298.7	535.5	110.4	102.9	0.914	2.912	10.04	0.026	0.137	0.018	0.001

*A. cruentus*												

SDU	3.368	796	422.5	173.6	106.5	0.462	3.402	48	0.025	ND	0.024	ND
CS	3.913	323.2	576.8	79.56	88.47	0.597	5.685	38.22	0.037	ND	0.022	ND

SDU: sun-dried and unsliced, CS: cooked and sliced, and ND: not detected.

**Table 3:** Proximate composition (g/100 g dry matter) of protein of reference (egg white) and differentially processed *A. hybridus* and *A. cruentus*.

Diet	Dry matter	Crude protein	Crude lipid	Crude fibre	Ash
Egg white	89.95 ± 0.99^ab^	80.05 ± 0.85^a^	1.07 ± 0.74^c^	1.73 ± 0.20^e^	6.89 ± 0.05^c^

*A. hybridus*					

SDU	89.58 ± 0.025^ab^	30.95 ± 0.07^dc^	3.80 ± 0.39^a^	6.02 ± 0.65^d^	15.43 ± 0.07^a^
CS	90.38 ± 0.08^a^	31.79 ± 0.04^c^	1.21 ± 0.03^c^	14.00 ± 0.08^a^	10.45 ± 0.12^bd^

*A. cruentus*					

SDU	88.487 ± 0.1^b^	32.22 ± 0.02^b^	1.64 ± 0.09^bc^	8.76 ± 0.15^c^	9.67 ± 0.04^d^
CS	90.02 ± 0.8^ab^	25.65 ± 0.01^e^	2.58 ± 0.07^b^	12.18 ± 0.66^b^	9.27 ± 0.60^d^

Results are expressed as mean ± standard deviation. Means in the same column followed by the same letters are not significantly different at 5%. DI: egg white (standard protein), SDU: sun-dried and unsliced, and CS: cooked and sliced.

**Table 4:** Growth performance and protein quality of rats fed with diets containing processed *A*. *hybridus* and *A*. *cruentus *leaves.

Parameters	Experimental diets					
	Standards		*A. hybridus*-based diets		*A. cruentus*-based diets	
	D0	DI	DII	DIII	DIV	DV
Food intake (g)	21.92 ± 2.92^c^	68.56 ± 9.04^a^	54.70 ± 5.35^b^	73.46 ± 5.57^a^	RNA	31.63 ± 5.77^c^
Protein consumed (g)	RNA	6.74 ± 1.06^ab^	5.47 ± 0.53^b^	7.34 ± 0.55^a^	RNA	3.16 ± 0.57^c^
weight gain (g)	−2.80 ± 0.83^d^	24.4 ± 3.71^a^	6.00 ± 1.58^c^	13.60 ± 1.67^b^	RNA	−2.25 ± 2.06^d^
Feed efficiency (g/g)	RNA	2.78 ± 0.41^b^	9.57 ± 2.47^a^	5.50 ± 1.13^b^	RNA	RNA
Faeces weight (g)	2.20 ± 0.44^d^	7.40 ± 1.67^c^	14.00 ± 1.00^ab^	16.90 ± 1.94^a^	RNA	11.75 ± 1.70^b^
EFU (%)	RNA	36.5 ± 5.30^a^	10.93 ± 2.39^c^	18.68 ± 3.18^b^	RNA	RNA
PER	RNA	3.65 ± 0.53^a^	1.09 ± 0.23^c^	1.86 ± 0.31^b^	RNA	RNA
NPR	RNA	3.27 ± 0.14^a^	0.63 ± 0.23^c^	1.52 ± 0.31^b^	RNA	RNA
TD (%)	RNA	89.84 ± 7.47^a^	84.42 ± 8.94^a^	92.00 ± 8.43^a^	RNA	57.23 ± 7.06^b^

Results are expressed as mean ± standard deviation. Means in the same line followed by the same letters are not significantly different at 5%. D0: protein-free diet, DI: egg white (standard protein), DII: sun-dried and unsliced *A. hybridus*, DIII: cooked and sliced *A. hybridus*, DIV: sun-dried and unsliced *A. cruentus*, and DV: cooked and sliced *A. cruentus*. TD: true digestibility, FE: feed efficiency, EFU: efficiency of food utilization, PER: protein efficiency ratio, NPR: net protein ratio, and RNA: results not available. All the group IV animals died before the end of experimental period.

**Table 5:** Biochemical parameters and haematological indices of rats fed with different processed *A. hybridus *and *A. cruentus* meal.

Parameters	Experimental diets				
	Standard	*A. hybridus*-based diets		*A. cruentus*-based diets	
	DI	DII	DIII	DIV	DV
AST (U/L)	105.30 ± 6.10^a^	52.50 ± 1.61^b^	35.53 ± 2.70^c^	RNA	35.04 ± 0.83^c^
ALT (U/L)	27.25 ± 0.59^a^	17.85 ± 0.10^c^	21.00 ± 1.01^b^	RNA	21.29 ± 0.80^b^
Creatinine (mg/dL)	1.41 ± 0.64^a^	0.99 ± 0.26^a^	1.24 ± 0.31^a^	RNA	0.58 ± 0.38^a^
Urea (mg/dL)	34.01 ± 0.58^d^	38.81 ± 1.09^c^	43.37 ± 3.72^b^	RNA	114.10 ± 0.70^a^
Albumin (g/dL)	4.07 ± 0.11^a^	3.08 ± 0.19^b^	3.08 ± 0.19^b^	RNA	3.44 ± 0.79^ab^
WBC (10^3^/*μ*L^−1^)	3.50 ± 2.32^a^	4.24 ± 1.41^a^	6.14 ± 2.48^a^	RNA	2.90 ± 1.37^a^
LYM (%)	84.24 ± 1.80^a^	70.72 ± 4.95^b^	76.80 ± 4.92^ab^	RNA	69.27 ± 5.15^b^
MID (%)	5.46 ± 0.34^c^	11.38 ± 2.95^b^	9.30 ± 0.95^b^	RNA	16.30 ± 2.55^a^
GRAN (%)	3.95 ± 1.15^b^	17.9 ± 2.48^a^	16.73 ± 6.65^a^	RNA	13.73 ± 2.95^a^
RBC (10^6^/*μ*L)	5.31 ± 0.94^a^	4.66 ± 2.68^a^	5.30 ± 1.13^a^	RNA	6.69 ± 1.05^a^
HB (g·dL^−1^)	11.46 ± 1.91^a^	12.94 ± 1.06^a^	13.76 ± 1.83^a^	RNA	15.2 ± 2.25^a^
HCT (%)	32.08 ± 4.06^a^	33.7 ± 2.37^a^	34.46 ± 3.79^a^	RNA	44.27 ± 1.70^b^
MCV (fL)	60.96 ± 3.46^ab^	58.38 ± 4.64^b^	68.28 ± 3.92^a^	RNA	66.3 ± 5.11^ab^
MCH (pg)	21.56 ± 0.92^a^	21.80 ± 1.36^a^	24.20 ± 4.80^a^	RNA	22.67 ± 0.23^a^
MCHC (g·dL^−1^)	35.64 ± 2.69^a^	37.50 ± 0.95^a^	36.33 ± 2.07^a^	RNA	34.93 ± 5.67^a^
PLT (10^3^/*μ*L)	581.20 ± 4.49^a^	436.40 ± 5.22^d^	486.80 ± 4.14^c^	RNA	13.00 ± 2.64^b^
MPV (fL)	7.78 ± 1.51^a^	8.12 ± 0.53^a^	7.92 ± 0.50^a^	RNA	7.80 ± 0.61^a^
PCT (%)	0.41 ± 0.22^a^	0.35 ± 0.28^a^	0.37 ± 0.10^a^	RNA	0.39 ± 0.11^a^

Results are expressed as mean ± standard deviation. Means in the same line followed by the same letters are not significantly different at 5%. DI: egg white (standard protein), DII: sun-dried and unsliced *A. hybridus*, DIII: cooked and sliced *A. hybridus*, DIV: sun-dried and unsliced *A. cruentus*, and DV: cooked and sliced *A. cruentus*. WBC: white blood cell, LYM: lymphocytes, MID: mid-cells, GRAN: granulocyte, RBC: red blood cell, HB: haemoglobin, HCT: haematocrit, MCV: mean cell volume, MCH: mean cell haemoglobin, MCHC: mean cell haemoglobin concentration, PLT: platelets, MPV: mean platelet volume, PCT: plateletcrit, and RNA: results not available. All the group IV animals died before the end of experimental period.
